# The “4 plus 2” rituximab protocol makes maintenance treatment unneeded in patients with refractory ANCA-associated vasculitis: A 10 years observation study

**DOI:** 10.18632/oncotarget.18120

**Published:** 2017-05-23

**Authors:** Dario Roccatello, Savino Sciascia, Daniela Rossi, Mirella Alpa, Carla Naretto, Massimo Radin, Roberta Fenoglio, Simone Baldovino, Elisa Menegatti

**Affiliations:** ^1^ Department of Clinical and Biological Sciences, Center of Research of Immunopathology and Rare Diseases, Coordinating Center of the Network for Rare Diseases of Piedmont and Aosta Valley, Department of Clinical and Biological Sciences, University of Turin, Turin, Italy; ^2^ Nephrology and Dialysis Unit, S. Giovanni Bosco Hospital and University of Turin, Turin, Italy

**Keywords:** rituximab, systemic vasculitis, ANCA-associated vasculitis, granulomatosis with polyangiitis, microscopic polyangiitis, Immunology and Microbiology Section, Immune response, Immunity

## Abstract

**Background:**

ANCA associated vasculitides (AAV) often present with a chronic relapsing course. Relapse leads to increased immunosuppressive exposure and consequent toxicity. While two randomized controlled trials have shown rituximab (RTX) to be the most effective induction treatment in patients with relapsing disease, the optimal treatment duration and RTX dose remain debated. Whether to administer a maintenance dose to every patient, at a fixed time interval or on the basis of B cell count and ANCA titre or only when disease manifestations do occur is still debated as well.

**Methods:**

11 patients (5 with granulomatosis with polyangiitis, 4 with microscopic polyangiitis-MPA-, and 2 with eosinophilic granulomatosis with polyangiitis -EGPA-) intolerant or refractory to conventional therapies including cyclophosphamide were enrolled. All patients received the so called “improved 4+2” RTX scheme as a rescue therapy. Following RTX administration, immunosuppressive drugs were rapidly tapered and no immunosuppressive maintenance therapy had been given.

**Results:**

After a mean follow-up of 85 months since the “4+2” RTX protocol: four out of 11 patients (37%, 1 EGPA and 3 MPA, all MPO-positive) remained in remission after one cycle of “4+2” RTX infusion protocol with no relapse for a median 66 months [60–108]). Seven relapsing patients were re-treated once with RTX (again as monotherapy with the same protocol) after a median of 54 months (24-96). Following re-treatment, they again showed complete remission over a median of 32 months (12-96) of observation.

**Conclusion:**

In one of the longest-term observation (85 months) studies, sustained clinical remission without immunosuppressive maintenance therapy (and a negligible dose of prednisone since the 5^th^month) was obtained by the “4 + 2” RTX infusion protocol in patients with ANCA-associated vasculitis intolerant or refractory to conventional therapy.

## INTRODUCTION

Granulomatosis with polyangiitis (GPA), microscopic polyangiitis (MPA), and eosinophilic granulomatosis with polyangiitis (EGPA) are small-vessel vasculitides associated with antineutrophil cytoplasm antibodies (ANCA) [1]. Classification has been historically performed according to the clinical phenotype together with ANCA positivity or histological confirmation [2], although a recent genome-wide association (GWAS) study demonstrated that, from a genetic perspective, ANCA-associated vasculitis (AAV) are best defined by ANCA specificity rather than clinical features [3]. Disease severity ranges from mild manifestations to life- or organ-threatening forms. AAV are characterized, particularly in the proteinase 3 (PR3)-associated group [4], by a chronic relapsing course leading to repeat exposure to immunosuppression with increased adverse events and organ damage [5].

Rituximab (RTX), an anti-CD20 monoclonal antibody, is an effective induction treatment of AAV in both newly diagnosed and relapsing patients [6–8]. The efficacy of repeat dose RTX as maintenance therapy has been currently debated in both retrospectively [9, 10] and prospectively studies [10]. However, a number of issues remain unmet, including the number/yearly and amounts of repeat dose RTX, and duration of maintenance treatment [11]. Whether or not redosing should be given at a fixed time interval or driven by rising of ANCA titre and B cell count or symptoms, or both, remains to be established. Moreover, the long-term outcome after discontinuation of RTX treatment is still unknown.

We have previously shown a favorable outcome of 11 patients with refractory AAV treated with a “4+2” RTX protocol [12]. RTX was administered after unsuccessful or not tolerated cycle(s) of cyclophosphamide in 9 out 11 patients. This“4+2” protocol (so called “improved protocol” [12]) had been generally well tolerated, but its long-term safety profile has to be demonstrated in AAV.

We are now reporting our 10-years’ experience with these 11 prospectively enrolled patients. Since our treatment policy was not to repeat RTX administration unless patients showed definite signs of clinical relapse, our data provide some evidence that at least in non-granulomatous or MPO-associated ANCA vasculitis, a maintenance therapy following the 4+2 RTX protocol is unneeded.

## RESULTS

The mean duration of disease and follow-up after “4+2” RTX protocol was 119 months (range 60-189 months) and 85 months (range 45-132 months), respectively.

Long term clinical and laboratory parameters before and after the “4+2” RTX protocol are shown in Table 1. Significant decreases (baseline mean value; 3 months, 1 year, 3 year, end of follow-up, *p* < 0.05) were found in the mean levels of ESR, ANCA, proteinuria/24h, IgG, IgM, and C-reactive protein.

**Table 1:** Clinical and laboratory parameters before and after the “4+2” Rituximab protocol

	Baseline (mean value±SD)	3 months	1 year	3 year	End of follow-up
CRP (mg/dl)	7.2±3.1	2.1±2.5	0.5±1.1	0.8±1.0	0.4±1.2
ESR mm/h	56 ±46	21±18	18±11	16±7	11±5
ANCA (UI/l)	25±15	12±7	5±6	9±7	5±6
Proteinuria (g/24h)	1.2±0.6	0.47±0.4	0.26±0.4	0.40±0.4	0.21±0.3
IgG (mg/dl)	976±123	715±65	698±87	701±57	681±98
IgM (mg/dl)	147±76	95±41	89±23	75±65	68±43
BVAS (mean)	22	4	3	0	0

ANCA, anti-neutrophil cytoplasmic antibodies (cut-off 10 IU/l); CRP, C-reactive protein.

Mean serum creatinine values were 1.49 mg/dl (0.6-2.4) at baseline, 1.07 (0.6-1.8) after 3 months, 1 year: 1.06 mg/dl; 3 year: 1.2 mg/dl and 1.09 mg/dl (0.6-1.6) at end of follow-up. The “4+2” RTX protocol resulted in a significant decrease of mean BVAS (baseline: 22; 3 months: 4; 1 year: 3; 3 year and end of follow-up: 0). Constitutional symptoms, including arthralgia, weakness, and fever, disappeared in all the previously affected patients, while large necrotizing skin ulcers and polyneuropathy gradually resolved in 9 months.

All patients had complete peripheral blood-B cell depletion after the first “4+2” RTX protocol. The CD20+ B cells were detectable in the circulation after a mean of 11.5 months (9-19 months). Of note, at 36 months, CD20 cell number was still lower than baseline (*p* < 0.01).

Patients were not given any further immunosuppressive maintenance therapy after “4+2” RTX protocol, and oral prednisone was tapered to 5 mg/day by the end of the 3rd month after RTX.

**Figure 1:**
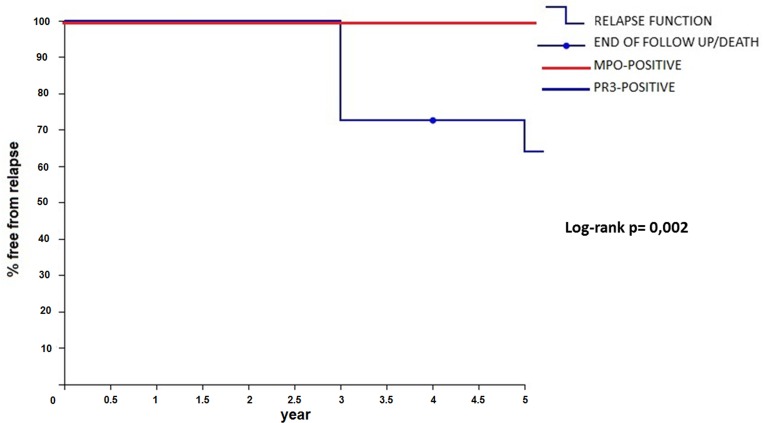
Relapse-free survival after rituximab (RTX) treatment stratified by MPO/PR3 positivity

Time free from disease after the first RTX treatment stratifying patients for ANCA profile is shown in Figure 1.

We failed to retrieve any independent predictors of AAV relapse after multivariate analysis, probably due to small sample size.

Seven out of 11 patients remained in remission for at least 60 months after one cycle of “4+2” RTX, and 4 did not experience any relapse at all, despite the absence of maintenance immunosuppressive therapy. Of note, all four out of 11 patients (37%, 1 EGPA, 3 MPA) that remained in remission after one cycle of “4+2” RTX protocol, with no relapse (median 66 months [60–108]) were MPO-positive. In the 7 relapsing patients (4 within and 3 after 5 years of follow-up) a second cycle of infusion of RTX alone was given because of worsening of clinical settings. Those patients showed a complete clinical response after the second cycle with normalization of the clinical and serologic profile. All 4 patients relapsing in the first 5 years of follow-up after the “4+2” RTX cycle were PR3 positive and relapses were seen after a median time of 36 months (range 36-60). Overall, the 7 relapsing patients were re-treated with RTX (again as monotherapy) after a median of 54 months (36-96). Six months after treatment for relapse, 6 of 7 patients (86%) were in complete remission, while 1 (14%) patient with EGPA experienced a minor relapse, requiring small doses of mycophenolate mofetil (MMF) as maintenance therapy at the dose of 500 mg/day, which was withdrawn 7 months after RTX administration. Following re-treatment, they again showed complete remission over a median of 32 months (12-96) of observation after a second cycle of RTX. There were 2 deaths (all due to cardiovascular causes - mean age of patients 79.2 years) after a median of 132 and 75 months after their first RTX cycle.

## DISCUSSION

AAV, especially cANCA-related vasculitides, often presented with relapsing course. Relapse leads to increased immunosuppressive exposure and toxicity. Two randomized controlled trials have shown RTX to be effective in inducing remission inpatients with newly diagnosed and relapsing disease. However, the optimal maintenance treatment is widely debated.

The “4 + 2” RTX protocol was used in the present study. This scheme was associated with a delayed occurrence of relapses with a complete discontinuation of steroids and immunosuppressive drugs [12]. This is the main interest of this protocol: the possibility to avoid the adverse effects related to the use of steroids and standard immunosuppressants, assuring a long-lasting remission. Besides, this scheme is also useful in low-compliant patients, and avoids hospitalization.

In a recent study, Alberici et al. [8] showed that relapses started to occur as early as 6 months after the last RTX infusion, but were more common between months 10 and 24. In their study, the use of RTX given at 1 g every 6 months for 24 months induced long-lasting remission of > 24 months in 50% of patients, while for the remaining RTX was able to keep the disease inactive while ongoing dosing is employed. At least in our hands, a single cycle of “4+2” Rituximab protocol was able to maintain a stable remission in more than 60% of the patients for at least 5 years.

When focusing on risk factors for relapses, several studies have shown that relapses are much more frequent in patients with PR3-AAV than in those with MPO-AAV, and PR3-ANCA positivity is often the most important risk factor for relapses in multivariate analyses [13–17]. A retrospective study found that the risk of relapse was particularly high if PR3-ANCAs persisting after induction therapy with cyclophosphamide [16], but this observation was not confirmed in a subsequent prospective study [17]. We observed that all 4 patients relapsing in the first 5 years of follow-up after the first “4+2” Rituximab cycle were PR3 positive. In our study, probably due to the small sample size, no determinant other than PR3-positivity was found to be associated to a higher risk of relapse in the first 5 years. Whether serial measurements of ANCAs (as well as peripheral blood CD20+B-cells) could be used to guide the frequency of re-treatment with rituximab as opposed to fixed-interval re-dosing [18] is currently under investigation in a prospective trial (MAINRITSAN 2 study) [19].

Due to the delayed onset of relapse, our experience suggests that the policy of monitoring patients could be better than to administer fixed doses of RTX whatever the clinical assessment, at least using our induction protocol.

In brief, the novelty of this open single-center study relies on the extremely long-term follow-up and on the discontinuation of steroid- and immunosuppressive drugs following the 4+2 RTX protocol. These results confirm our observations in other immune-mediated diseases [20–23]. When compared to other regimen suggesting the use of RTX on a fixed scheme, our findings support the choice of a treatment-to-target approach to achieve an adequate degree of B-cell depletion and a clinical response in AAV. The efficacy of the 4+2 dose RTX protocol is probably related to a more effective tissue depleting activity when compared to the standard scheme, providing a more prolonged clinical remission.

## LIMITATIONS OF THE STUDY

The main limitations of our study are the open non-blinded nature and the limited number of enrolled subjects. However, those are counterbalanced by the facts that our data are supported by a very long term follow-up in a cohort of real-life AAV patients and that “4+2 RTX protocol” allows avoidance of standard maintenance immunosuppression and has a substantial steroid-sparing effect.

## CONCLUSION

In one of longest follow up study (45-132 months), sustained clinical remission without immunosuppressive maintenance therapy (and a negligible dose of prednisone since the 3^rd^month) was obtained by a 4 + 2 infusion protocol of RTX in a selected sample of patients with ANCA-associated vasculitis intolerant or refractory to conventional therapy.

## MATERIALS AND METHODS

### Patients

Eleven patients with ANCA-associated vasculitis, 5 women and 6 men, 5 with granulomatosis with polyangiitis (GPA), 4 with microscopic polyangiitis (MPA), and 2 with eosinophilic granulomatosis with polyangiitis (EGPA), mean age at the time of fist RTX cycle 67.5 years (range 45-81), were deemed eligible for RTX therapy. Diagnosis of AAV was done according to the Chapel Hill Consensus Conference definitions [1] and reviewed according to the modified version [24]. Demographic data, clinical presentation and previous treatments are described in details elsewhere [12].

In brief, 7 patients presented with renal involvement: 6 had a biopsy-proven necrotizing extracapillary paucimmune glomerulonephritis, 1 case did not undergo renal biopsy because of concomitant anti-coagulation therapy due to the presence of antiphospholipid antibodies. Sinus involvement and lung nodules were detected in 5 cases, retro-orbital granulomata in 2 patients. Biopsy-proven leukocytoclastic vasculitis and large necrotizing skin ulcers were present in 2 cases. Polyneuropathy was observed in 7 patients, while arthralgia and weakness were found in 9 cases, and fever in 6. Six patients (3 GPA 2 MPA and 1 EGPA) were found to be resistant to cyclophosphamide (CYC). One more GPA patient was in a relapsing phase albeit an elevated cumulative dose of CYC. Another GPA patient had a history of severe CYC-induced leukopenia when addressed to our centre from another hospital because of a life-threatening disease. A MPA patient could not be treated with CYC because of a sudden pancytopenia after 1 week of oral treatment at the conventional dose (2 mg/kg). One MPA and 1 EGPA CYC-naive patient refused to accept the risks of infertility related to CYC treatment.

### Protocol

RTX was administered intravenously at a dose of 375 mg/m 2 on days 1, 8, 15 and 22. Two more doses were administered 1 and 2 months after the last administration [23].

### Response to treatment

Response was evaluated by assessing the changes in clinical signs and symptoms and laboratory parameters. Major relapse was defined as reappearance or worsening of disease as measured with an increase of at least 1 point of BVAS when compared to the score value obtained after previous treatment and involvement of at least one major organ. Minor relapse was defined as worsening of disease with mild clinical symptoms associated with re-appearance of ANCA together with CD20 repopulation.

The Birmingham Vasculitis Activity Score (BVAS) was calculated at study entry, monthly for the first 6 months and every six month during the follow-up or in case of relapse [25].

### Statistical analysis

For comparison of variables at baseline and follow-up, Student's *t*-test was used for continuous and Fisher's test for categorical variables, as appropriate. Correlations were calculated and significance determined by. Multivariable logistic regression analysis was used to identify any independent predictors of AAV relapse. Kaplan-Meier hazard plots were constructed for time to time to AAV relapse. For these analyses, SPSS (IBM Corporation, NY, USA) software program was used. *p* < 0.05 was considered significant.

This study was performed according to the local rules of off-label therapy in Piedmont Region (Northwest Italy).
